# Process evaluation of a web-based intervention aimed at empowerment of disability benefit claimants

**DOI:** 10.1186/1472-6947-11-10

**Published:** 2011-02-16

**Authors:** David Samoocha, Ingrid AK Snels, David J Bruinvels, Johannes R Anema, Wojtek Kowalczyk, Allard J van der Beek

**Affiliations:** 1 Department of Public and Occupational Health, EMGO Institute for Health and Care Research, VU University Medical Center, Amsterdam, The Netherlands; 2 Research Center for Insurance Medicine AMC-UMCG-UWV-VUmc, The Netherlands; 3 Department of Computer Science, VU University, Amsterdam, The Netherlands

## Abstract

**Background:**

The objective of this process evaluation study was to gain insight into the reach, compliance, appreciation, usage barriers, and users' perceived effectiveness of a web-based intervention http://www.wiagesprek.nl. This intervention was aimed at empowerment of disability claimants, prior to the assessment of disability by an insurance physician.

**Methods:**

Reach was determined by registering claimants exposed to the study's invitation brochures, and by comparing trial participant characteristics with non-participants and nationwide claimant data. Compliance was registered by analyzing weblogs, which were automatically collected during the period of the trial. This made it possible to analyze individual use of the intervention. Appreciation, usage barriers, and users' perceived effectiveness were assessed using an online questionnaire that was sent to participants from the intervention group, 6 weeks after enrolment.

**Results:**

Only 9% of the target population enrolled in the internet program. Because of selective enrolment, more females, higher educated claimants, and less ethnical minorities were reached. Compliance was ambiguous: out of the 123 participants randomized into the intervention group, a significant proportion (33%) did not use the intervention at all, while, at the same time, many participants (32%) used the intervention for more than two hours (i.e. in approximately two weeks). Overall satisfaction with the intervention was good. Claimants perceived the intervention most effective in increasing knowledge, while also a fair amount of users perceived the intervention effective in gaining right expectations or being able to communicate better with their physician.

**Conclusions:**

The uptake of the intervention http://www.wiagesprek.nl was disappointing. Specifically, the poor reach and compliance of the intervention resulted in a small proportion of the target population using the intervention as intended. Improvements in the implementation process are desirable to increase the reach and compliance and, thereby possibly, the impact of the intervention.

**Trial registration:**

NTR-1414

## Background

Effective patient-physician communication is considered to be an essential aspect of high quality health care [1,2]. For some years, the trend has been to put more emphasize on the patients role in the patient-physician interaction [3]. Evidence exists that patients' active participation during the medical interview is associated with, for example, better health outcomes [4] and patient satisfaction [5]. Accordingly, an increasing amount of interventions are aimed at patients prior to consultation visits, which vary from information provision, helping patients to formulate questions to ask their physicians, to role-playing exercises that increase attention to behavioural styles [6,7]. Although the vast majority of these interventions are applied in primary care, it is possible that the benefits of activating patients by empowering interventions prior to consultation visits can be of importance in other fields of health care. A field that can possibly benefit from patient empowerment is insurance medicine.

Through social insurance, employees have the possibility to claim a social insurance pension when they are losing (part of) their income due to work disability. The process of evaluating work disability claims, which is of great societal and financial importance, is significantly being determined by a disability assessment interview, in which insurance physicians have to determine the patients work capacity and functional limitations [8]. This specific patient-physician interaction has some characteristics that can complicate the communication between the patient (or claimant) and the physician, which have been described elsewhere [9,10].

In an attempt to overcome these difficulties, and to enhance the claimant-physician communication, an intervention was developed [9]. Since the Internet has the potential to reach a large audience at a low cost [11], it was chosen to deliver this intervention online. As such, the web-based intervention http://www.wiagesprek.nl was available for disability benefit claimants in the Netherlands in the period January 2009 until September 2009. Beforehand, it was expected that a large proportion of disability benefit claimants would use this intervention, especially because the Dutch population is known to have a high accessibility to the Internet. Approximately 90% of the general population in the Netherlands has access to the Internet (while the EU average is 68%) [12].

The web-based intervention http://www.wiagesprek.nl, aimed at empowerment of disability claimants, is currently being evaluated in a randomized controlled trial. In order to get more insight into how the intervention was implemented and received, what its strengths and weaknesses were, and under what conditions it was delivered, a process evaluation was conducted parallel to the randomized trial.

In the past years, the conduct of process evaluations alongside randomized controlled trials has been recommended, because they can facilitate the interpretation of the findings [13]. For example, a process evaluation can shed light on: (1) whether the intervention was delivered as intended (i.e. compliance, satisfactions, and experiences); and (2) the success and failures of the intervention program [14-16]. Moreover, the information obtained from a process evaluation can be used to further improve the intervention [15], and to enable the transition of research evidence into health practice [17].

The aim of the process evaluation described in this article was to assess the reach, compliance, appreciation, usage barriers, and users' perceived effectiveness of a web-based intervention http://www.wiagesprek.nl that was aimed at empowerment of disability claimants. By evaluating these process evaluation indicators, insight can be gained into factors that have an influence on the implementation of the intervention, which can assist future implementation plans.

## Methods

This process evaluation is part of a randomized controlled trial on the effectiveness of the web-based intervention http://www.wiagesprek.nl: an intervention aimed at empowerment of disability benefit claimants prior to visiting an insurance physician for assessment of disability. The Medical Ethics Committee of the VU University Medical Center approved the study protocol.

### Description of the web-based intervention

The web-based intervention http://www.wiagesprek.nl was accessible approximately 1-2 weeks prior to the claimants' disability assessment. Content of this intervention was developed by using the Intervention Mapping protocol [18]. By applying this protocol a final version of the intervention was created, which consists of:

(1) Five interactive modules (estimated walk through time: 120-150 minutes), in which claimants are prepared for their disability assessment step-by-step, based on an empowerment approach. This approach focused on increasing knowledge about Dutch disability legislation and disability benefit procedures, skill gaining (question asking, negotiating) to improve patient-physician communication, promoting active participation during the disability interview, increasing claimants' awareness of their functional limitations with respect to work, and adapting expectations about disability assessment outcomes. Throughout the modules, participants were asked to fill in short assignments, such as knowledge quizzes or, for example, an assignment aimed at taking along a personal health record to the interview.

(2) General information and features concerning absenteeism from work, such as social security law arrangements, explanation of disability assessment procedures, return to work information, two videos of personal experiences of people who underwent disability assessment procedures, how to cope with disease and work disability, and links to other related websites.

(3) A forum in which participants are able to interact with other claimants on issues such as coping with disease or exchanging experiences concerning disability assessments.

A more detailed description of the intervention and its development has been published elsewhere [9].

### Study population

Participants were claimants for a disability pension according to the Dutch *Work and Income Act (WIA)*, which can be claimed after being sick-listed for 104 weeks. All disability claimants were recruited approximately 1-2 weeks prior to their appointment for disability assessment by an insurance physician. Recruitment took place at three different offices (Leiden, The Hague and Rotterdam) of the Dutch Workers Insurance Authority (UWV). UWV is an organization in the Netherlands that is responsible for evaluating disability claims. Together with a standard invitational letter from UWV, claimants received a study information brochure, which directed them to an online application form. This application form included questions concerning the study's in- and exclusion criteria and an informed consent. Claimants were considered eligible to participate in the study if they had adequate knowledge of the Dutch language, and had an email address. Recruitment took place over a 9-month period (January 2009 - September 2009). Claimants received a 10 euro voucher if they applied to participate and completed the baseline questionnaire.

### Data Collection

#### Reach

Reach is defined as the absolute number, proportion and representativeness of subjects who participated in the trial. To determine reach, the following steps were undertaken:

(1) During the process of the trial, the total number of invitation brochures sent to claimants was registered.

(2) The number and some characteristics of non-participants and reasons for non-participation were registered. This was done by attaching a reply envelope to the study's invitation brochure, meant to be filled out by claimants who were not interested or able to participate. Non-participants' age, gender, country of birth, and disease type were noted on these envelopes.

(3) Nationwide, representative data were collected from all UWV departments in the Netherlands in order to obtain information on main characteristics (age, gender, type of disease) for all workers claiming disability during the period of the trial. Additionally, these data were also retrieved from the three UWV offices that participated in the trial in order to determine the representativeness of these offices.

#### Compliance

Compliance refers to the extent to which the intervention was used and is the opposite of non-usage attrition, a term often used in describing web-based program use [19]. User authentication (obtaining username and password at the beginning of every session) made it possible to register activity for each individual participant. With the appropriate scripting language (PHP: Hypertext Preprocessor) every activity on the website was registered in a MySQL database. This database contained weblogs with every row of the database containing the participants' ID number, page number visited, time stamp (start and end time), and session number. To limit overestimation of activity time, a timer was built in the system, which stopped time registration when a participant was not active (scrolling, click or mouse movement) for a period of 8 minutes.

With data from the weblogs it was possible to calculate the following variables for each participant: total time of intervention use, amount of unique page views, total number of sessions, amount of clicks, and time spend on each module. Furthermore, for each page it was possible to collect statistics in order to gain insight in the most used components of the program. Variables that were distinguished were: amount of unique visitors and total time on page. Calculating weblogs into user and page statistics was done using MATLAB version 7.3.

Besides using weblogs, from a separate MySQL database, participant data on which modules were started and finished were distracted, as well as activity on the forum by means of amount of posts and post views.

### Other Process Evaluation Indicators

Data from the following process evaluation indicators were collected by means of an online questionnaire that was sent to participants of the intervention group six weeks after their disability assessment (approximately 7-8 weeks after study enrolment): appreciation of the program, usage barriers, perceived effectiveness, and suggestions for improvement.

#### Program Appreciation

Appreciation of the program was assessed 4-fold:

(1) participants were asked to give a generic grade (range: 1-10) for the intervention,

(2) participants were asked to rate their appreciation of each of the five modules, also by means of a generic grade (1-10), and answer the statement "this module was helpful to me" on a 5-point Likert scale (ranging from totally do not agree (1) to totally agree (5)),

(3) we asked participants what they thought were the most useful components of the program, and

(4) statements concerning the appreciation of the program were presented to participants, on which they could respond by indicating their level of a agreement on a 5-point Likert scale (ranging from totally do not agree (1) to totally agree (5)). Example statements were: "I found useful information on the website" or "I didn't like the *look and feel *of the website".

#### Usage Barriers

We asked the least active users (0-60 minutes of total web activity) for what reason(s) they did not -or barely- used the program. Answer categories were: 1) I could not find information that I was looking for on the website, 2) I did not feel the need to prepare myself extensively for my upcoming disability assessment interview, 3) personally, the site did not appeal to me, 4) I did not have enough time to prepare myself, 5) I already prepared myself adequately before this website was brought to my attention, or 6) other reasons (open category).

#### Perceived Effectiveness

Participants were asked how effective they thought the website intervention was in order to: 1) be able to communicate more effectively with their insurance physician, 2) gain knowledge about disability assessment procedures and work disability legislation, 3) gain insight into what to expect from a disability assessment, 4) gain skills that were useful in the disability assessment interview, and 5) trigger them to start thinking about their lives after the disability assessment procedures.

#### Suggestions for improvement

We asked participants if they had suggestions to improve the program. We illustrate the results with some quotes providing qualitative insight into the opinions of the program users.

### Data Analysis

Quantitative data was analyzed by means of descriptive statistics. Differences between participants and non-participants were tested with t-tests and the Pearson Chi-Square test. In all analysis we used SPPS version 15.0.

## Results

### Reach

From the 2780 disability claimants who were approached for participation, 2329 (84%) claimants did not respond to the study's invitation, 95 (3.4%) did not meet the inclusion criteria, 91 (3.3%) were unwilling to participate, and 23 (0.8%) participants responded too late (i.e. their application was received after their appointment with the insurance physician). The remaining 242 participants (8.7%) were randomized to either the intervention group (n = 123) or the control group (n = 119). Baseline characteristics of the study participants and non-participants are shown in Table 1.

**Table 1:** Characteristics of claimants that: participated in the trial (column 1), did not participate in the trial but responded to the study's invitation (column 2), claimed disability in the year 2009 in one of the three participating UWV offices (column 3) and claimed disability in the year 2009 in one of all the UWV offices in the Netherlands (column 4).

	Participants (n = 242)	Non-Participants (n = 186)	Participating UWV offices (n = 6034)	All UWV offices (n = 37607)
**Mean (SD) age (years)**	48.66 (9.7)	48.23 (10.4)	45.53 (10.5)	45.58 (10.6)
**Female, %**	60.3	47.6	55.6	54.3
**Country of Birth, %**				
**The Netherlands**	86.8	73.1	unknown	unknown
**Education, %**				
**Lower**	26.4	unknown	unknown	unknown
**Middle**	47.9			
**Higher**	25.6			

Main reasons for not willing to participate were: not willing to fill out questionnaires (n = 31: 37%), lack of need to prepare for the disability assessment (n = 23: 27%), disability too severe in order to participate (n = 16: 19%), or other reasons (n = 14: 17%).

No clear differences between the three participating UWV offices and national data was found in age, gender and disease type, indicating that patients visiting these offices were comparable and representative to all claimants in the Netherlands, based on these three characteristics.

When comparing characteristics of trial participants with national data and non-participant data, it appeared that females and claimants born in the Netherlands were overrepresented in the trial, and that trial participants were slightly older than the average Dutch claimant.

### Compliance

**Figure 1:**
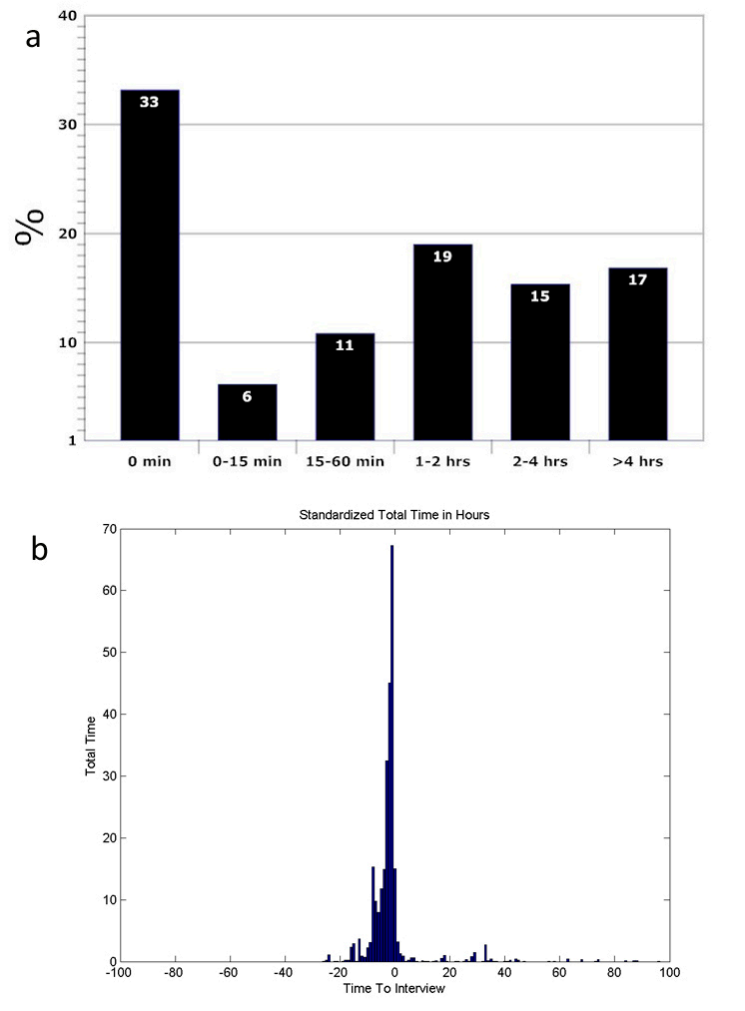
**Compliance: (a) percentage of claimants that spent [0 min], [0-15 min], [15-60 min], [1-2 hrs], [2-4 hrs] and [more than 4 hrs] on the intervention respectively, (b) Standardized to the date of the disability interview**. (X-axis: amount of days before (-) and after (+) the interview, Y-axis: cumulative amount of hours spent on a day by all claimants)

During the time of the trial the intervention http://www.wiagesprek.nl was accessed 329 times by the 123 participants of the intervention group. The average time these participants spent on the website was 115.3 minutes (SD 160), with an average of 2.7 sessions (SD 4.07), on average, 32.6 of the (in total) 91 unique pages were viewed (SD 31.5) and an average of 99.1 clicks (SD 127) were made. Interestingly, from all intervention group participants, 41 (33%) never logged on to the website after enrolment. On the other hand, 39 (32%) used the intervention more than two hours, and 27 (22%) finished all five modules. Figure 1(a) shows the percentage of participants that used the intervention for 0 minutes, 0-15 minutes, 15-60 minutes, 1-2 hours, 2-4 hours and more than 4 hours, respectively. When standardizing compliance to the date of the disability assessment, Figure 1(b) shows that most web activity found place the day before the assessment interview, and that there still remained some activity up until several weeks after the assessment.

With regard to the elements of the intervention that were most used, the distribution of web activity was as follows: from the, in total, 14020 minutes that participants spent on the intervention, 1345 minutes (9.6%) were spend on the pages with general information, 7837 minutes (55.9%) on the five modules, 3756 minutes (26.8%) on filling out the personal health record, 530 (3.8%) minutes on the calendar and diary, and 550 minutes (3.9%) on the forum. Interactivity on the forum was very low: although 68 participants viewed some posts that were started by the moderator, only six participants started a post by writing on their personal experience with their disability assessment and only one participant started a post in the question asking forum.

### Questionnaire

The response to the online questionnaire was 84% (n = 103). Reasons for not responding to the questionnaire were unknown.

#### Program Appreciation

Among participants from the intervention group that logged on at least once (n = 82), the generic grade that was given to the website intervention was 7.32 (SD 0.83). The most useful components mentioned were: pages with general information (mentioned by 82% of all participants), the five modules (63%), personal health record assignment (47%), links to other related website (18%), the forum (9%), and the assignment with the calendar and diary (9%).

Table 2 shows the appreciation, use and perceived benefit for each of the five modules.

**Table 2:** Module appreciation, use and perceived benefit among intervention users

Modules	Mean Time spent (min)	Number of Participants Started (Finished) Module	Grade	Module useful?				
				Totally not agree	Not agree	Neutral	Agree	Totally agree
**Module 1**	30.45	77 (53)	7.25	0%	0%	11%	72%	17%
**Module 2**	20.43	62 (55)	6.98	0%	0%	20%	62%	18%
**Module 3**	16.40	56 (45)	6.93	0%	0%	20%	64%	16%
**Module 4**	9.38	49 (39)	7.08	0%	2%	13%	67%	18%
**Module 5**	9.62	43 (36)	7.25	0%	3%	8%	81%	8%

With regard to the overall appreciation of the intervention, Table 3 shows claimants' answers to the statements concerning the content of the website. Most users found the information on the website useful (85%), clear and easy to understand (83%), and the website easy to use (69%). However, according to a substantial proportion of users (41%), the website contained too much information, and for some (14%) the loading of pages lasted too long.

**Table 3:** Appreciation of the intervention among the intervention users (n = 82)

Statements	Totally not agree (%)	Not agree (%)	Neutral (%)	Agree (%)	Totally agree (%)
I found useful information on the website	1	1	13	69	16
The information on the website was clear and understandable	1	1	14	63	20
I missed information on the website that I was looking for (-)	11	47	31	11	0
I find the website easy to use	3	6	23	56	13
The amount of information that you had to go through was too much	1	27	31	34	7
The links on the website are hard to find (-)	6	56	41	3	0
Loading pages lasted too long (-)	7	37	42	10	4
I didn't like the *look and feel *of the website (-)	4	39	51	6	0
Information on this website was not useful to me (-)	17	55	23	6	0
The language that was used was easy to understand	0	4	23	59	14
It was hard for me to work with this website (-)	17	58	23	1	1
I find the video's on the site helpful	1	6	55	33	4

### Usage Barriers

The most important reason for not using -or barely (0-60 min)- using the intervention program (n = 48) was the fact that some participants did not feel the need to prepare themselves for the interview extensively (n = 13: 28%). Other factors were that some participants already prepared themselves properly before hearing from the website (n = 9: 19%). Some reported that the time between enrollment and the interview was too short (n = 7: 14%). Less mentioned barriers were related to the intervention itself: two participants (6%) did not find information they were looking for on the website, and one participant stopped using the site because it did not appeal to her. Other reasons that were noted, were, for example: "preparing yourself doesn't make any sense: the outcome is already determined" (2x), "because of my disease I have difficulties with reading", or "I didn't expect to get a disability pension so I didn't prepare myself".

### Perceived Effectiveness

**Figure 2:**
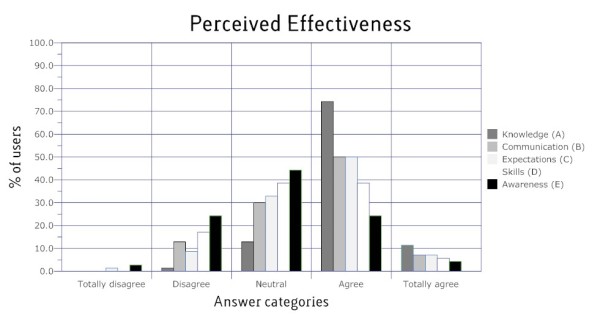
**Claimants' perceived effectiveness of the intervention on: (A) gaining knowledge, (B) being able to communicate better, (C) gaining right expectations, (D) improving skills and (E) gaining awareness**.

Figure 2 shows participants' perceived effectiveness of the intervention. According to the participants, the website was most effective in gaining knowledge (86% "agrees" or "totally agrees"). Furthermore, a fair amount of users perceived the intervention effective in gaining right expectations (58%) or being able to communicate better with their physician (57%). Less claimants found the intervention effective in gaining skills (44%) or in increasing awareness of their functional limitations with respect to work (30%).

### Suggestions for improvement

In total, 34 claimants (41% of the intervention users) gave suggestions for improvement. Many qualitative reactions from intervention users had overlap and were categorized into several themes:

#### Available time

Four claimants made comments concerning the available time for using the website prior to the disability assessment. For example, one claimant reacted:

"Please send the [study's] invitation earlier so you have more time to prepare yourself." Another claimant: "Make clear that you have to start preparing yourself on time. In my case, I started visiting the website one day before my disability interview. It was hard for me to go through all the information and modules in such a short time-frame."

#### Powerlessness

Some claimants felt that preparing yourself before a disability assessment did not make any sense and experienced an inability to have influence or to have control over the interview. According to these claimants, the outcome was already determined. For example:

"Put more emphasize on the fact that there is not a real conversation with you and the physician. Really, your arguments do not matter [..] the whole interview is so predetermined." Or: "Unnecessary waste of time and energy if you are trying to get your rights here."

#### Technical issues

A few claimants made comments about technical difficulties:

"I had some problems with playing the video's on the site." Or with the amount of information one had to read: "You have to do a whole lot of reading. This is very difficult for someone with an aphasia."

#### Language use

Two claimants experienced that the language that was used on the website was too simple:

"Maybe it's an idea to have two versions of the website: one like the present and one without the schoolish modules.[..] But it definitively prepared me well for the interview!" Another: "A little bit less childish language usage please."

Besides suggestions for improvement, many of the comments were positive, a few examples:

"I think the website is already complete as it is." Or: "Very clear and well-ordered website. My compliments!" And: "For me, the site contributed to an optimal interview with my physician."

## Discussion

The aim of this paper was to evaluate the implementation of the web-based intervention http://www.wiagesprek.nl, by describing its reach, compliance, usage barriers, appreciation and users' perceived effectiveness.

### Main Findings

#### Reach

The intervention's reach was low: only 242 out of the 2780 (± 9%) disability claimants to whom the study's invitation brochure was sent, participated in the trial. This low recruitment rate is a very common feature in online trials [20-22] which has serious consequences for the representativeness of a study sample [23]. Although the current study is unique regarding its purpose, study population and its setting, there are some comparable features with other studies when considering the low recruitment rate: First, the proportion of female claimants that participated in our trial was higher than the national mean (60% of the trial participants were female versus 54% of the national mean). This finding is in line with evidence from other online trials, which suggests that women generally exhibit more active information-seeking behaviour than men [23]. Second, ethnic minorities were under-represented in our trial. Although it was not possible to retrieve nationwide data on claimants country of birth, non-participant data showed that more than 25% of the claimants were not born in the Netherlands (versus 13% in our trial). Several reports that examine comparable populations confirm this and found around 20% of their investigated cohorts to consist of ethnic minorities [24,25]. Furthermore, it seems that a relatively high number of higher educated claimants took part of the trial, when comparing these data with other studies [24,25].

Although the intervention was initially intended for all claimants in the Netherlands, we can conclude that only a minority of claimants was interested in using it. Especially ethnic minorities, lower educated claimants, and male claimants seem to be less interested in using the intervention. In terms of recruitment, these findings are generally congruent with research on online trials, in which it seems that internet access patterns may create self-selection towards participants who are Caucasian and higher educated [22,26].

With regard to the reported reasons for non-participating in the trial, it was found that not having an Internet connection or email address was the main reason for not participating: 32% of all non-participants pointed out this argument. Other reasons were: not having the skills to use the Internet properly (21%) and not wanting to fill out questionnaires that were part of the research project (17%). Considering the reason of not having an Internet connection seems a bit odd, since most people in the Netherlands (about 90%) do have access to the Internet [12]. However, from a Dutch national survey it was additionally found that this high percentage of accessibility to the Internet can be much lower among subgroups that are frequently associated with the target population of our study, i.e.: lower educated, older, non-native, and disabled Dutch people [27]. This feature may consequently explain why these sub-groups (often not in the possession of a computer), were under-represented in the trial. To reach these groups, perhaps claimants should be enabled to use computers at, for example, UWV offices, the city hall, employment agencies or other public places.

#### Compliance

As one would expect from an online trial, not all participants used the intervention as intended. A large group of participants (33%) never logged on to the website after enrolment. This phenomenon of participants not using an intervention after study enrolment can be found in other web-based trials [28,29] and can be the result of, for example, technical problems or participants signing up just in order to receive enrolment incentives. In our trial, many non-users kept filling out follow-up questionnaires, which makes the latter reason implausible. A more plausible explanation for the high non-usage rate can be that claimants that filled in the study's online application form just very shortly before their disability assessment, received their username and password relatively late and, therefore, did not had the opportunity to use the intervention on time. In our trial, support for this finding was that 54% of all non-users signed up just one day before their disability interview, while this was 27% among the users of the intervention. Another important aspect can be that, although many people do have access to the Internet, computer literacy and computer skills are believed to be sometimes lacking among our target population. For example, only 19% of all Dutch people aged 55 years or older (39% of the participants in our trial were older than 55), knows how to send messages on a forum or chatroom [27]. For these subgroups, using a rather extensive website with several features and functionalities may be too complicated to use.

Despite the high non-usage rate, many participants used the intervention extensively. For example, 32% of all participants used the intervention more than two hours and a significant proportion (33%) of the participants that logged on at least once (n = 82) finished all five modules. The most mentioned barriers for using the intervention were: no need for an extensive preparation for the interview, already properly prepared for the interview and a lack of preparation time.

#### Appreciation and perceived effectiveness

Appreciation of the intervention was good. Participants that used the intervention rated the website with a 7.32 (range 1-10). A large proportion of the participants found the modules useful. Furthermore, the vast majority of the program users had the opinion that they found useful information on the website and that this information was easy to understand. However, some claimants found the amount of information on the site too extensive. With regard to the perceived effectiveness of the intervention, according to claimants, the intervention was most effective in increasing knowledge. Also, many claimants experienced the website as being helpful to improve communication with their physician and to create realistic expectations.

### Strengths and limitations

This study extensively collected data on process evaluation indicators. One of the major strengths of this study is that we used an accurate method to assess compliance with the intervention. As it is increasingly being stimulated and recommended to measure and report compliance in web-based research [19,30], in this trial, weblogs made it possible to register web activity for each participant individually and thereby give a reliable estimation of individual program exposure. This method of determining compliance is much more reliable than, for example, using self-reported program exposure data or simply registering if a certain component of an intervention was used or not [31]. In addition, we introduced a timer, which stopped individual time registration from the moment a participant was not actively using (scrolling, clicking or mouse movement) the website for a period of 8 minutes. This built-in timer minimalized possible overestimation of program exposure through eliminating the contribution of 'passive' time registration.

Another strength of the study was the availability of nationwide claimant data. With this data that was retrieved from UWV, it was possible to make comparisons between the trial participants and the target population, and thereby determining the interventions reach. Furthermore, the response to the online questionnaire measuring program appreciation, usage barriers and perceived effectiveness was satisfactory (84%). This made the outcomes of these variables have a high validity and reliability.

There were also some limitations in this study. First, there was a very high non-response within non-participants. Only 7.3% of all claimants that did not participate in the study returned response envelopes in which non-participants could fill out demographic data and reasons for not willing or being able to participate. Because of this high non-response, it is hard to generalize these data from non-participants. Every comparison made between participants and non-participants should, therefore, be interpreted with caution. Second, we could not retrieve national data on every demographic variable in order to make comparisons on a broad scale of variables. As such, we were not able to retrieve national data on average claimant education or country of birth. Comparisons made between participants and national data were solely based on age and gender. For the remainder of comparisons we used reports from other research which possibly may contain bias through a selective response. Finally, appreciation of the intervention could be overrated, because there is a chance that more satisfied claimants filled out the online questionnaire more often that dissatisfied claimants.

### Implications for Future Implementation

The process evaluation that has been conducted in this study gives insight into aspects involved in the implementation of the web-based intervention http://www.wiagesprek.nl. Besides the fact that this observed implementation helps interpreting the coming trial results, it also gives implications to further improve to process of implementation in order to enhance the impact of the intervention in the future. Most notably, this study shows that the interventions reach and compliance were disappointing aspects of the implementation process. With regard to the reach, it can be estimated that only about 9% of all disability claimants in the Netherlands will be interested in using the intervention. With a total of 38 thousand workers that claim disability annually, roughly, this means that about 3400 claimants will use the intervention every year. As can be distracted from our compliance data, it is estimated that of these 3400 claimants, about one third (around 1200 a year) will use the intervention intensively. With an estimated 3% (1200 out of 38000) of claimants using the intervention as intended, it can be concluded that the implementation strategy of the intervention was not very successful. Some aspects of the implementation process can therefore be adapted in the future. With regard to the timing of delivering the intervention it perhaps can be useful to make disability claimants aware of the website in an earlier stage before the disability assessment, although this would require a change in UWV's administrative processes. Furthermore, a more personal approach (e.g. by telephone or by face-to-face mediation) can help to improve the intervention's reach. In this case, administrative employees of UWV can, for example, contact claimants some time before the assessment as a reminder to use the intervention or as a personal assistant by giving help in using the website. Also, emphasizing claimants that they need to start their preparation for the assessment in an early stage can be useful, either communicated more explicitly on the website or in the application procedures. Although not asked to participants in this process-evaluation, it could be that claimants were in fear of using the intervention because of the possibility that data they filled out on the website would be used by UWV. As such, it could help to emphasize more that the intervention was delivered independently of UWV and that individual data was treated anonymously.

In summary, although the intervention was developed and intended for all disability claimants in the Netherlands, it seems that only a small subgroup of higher educated, Dutch-born, female claimants eventually used the intervention. When considering factors that are believed to influence the successes of an implementation [32], some additional suggestions can be made in order to possibly improve implementation in the future. Together with the already mentioned aspects, Table 4 sums up some possible suggestions to improve the uptake of the intervention by enhancing its reach and compliance.

**Table 4:** Suggestions for increasing the uptake of the intervention http://www.wiagesprek.nl

	Current	How to improve?
**Reach**	Low: 9% of all claimants will use the intervention. Under-representation of ethnic minorities, lower-educated and male claimants.	By adapting the Implementation strategy: - Start recruitment in an earlier stage, e.g. one month before the disability assessment - Independent delivery, through other channels than UWV, e.g. patient organizations - Invitations to use the intervention more directed at not-Dutch born claimants and lower educated claimants, e.g. by using multilingual brochures - Recruitment not only online, but by a more personal invitation, e.g. by phone - Increase perceived advantage, e.g. by adapting the content of the invitation brochures, experimenting with different contents, lay outs etc.
**Compliance**	Low: 33% of claimants that are interested to use the intervention, use the intervention as intended (more than 2 hours).	By adapting the Innovation itself: - To prevent that attrition leads to missing out relevant information, make a "a la carte" menu, in which claimants can choose what type of information they are interested in - Delete non-used or barely used components of the intervention, such as the forum, calendar/diary By adapting the Implementation strategy: - Start recruitment in an earlier stage, to give claimants that have logged on and started the program, enough time to finish the program

## Conclusions

The intervention discussed in this article was not delivered as intended. Only 9% of the target population enrolled in the Internet program. Because of selective enrolment, relatively more females, more higher educated claimants, and less ethnical minorities were reached. Compliance was ambiguous: a substantial proportion did not use the intervention at all, while, at the same time, many participants used the intervention extensively. It is estimated that of the target population exposed to the offer of using the intervention, only 3% will use the intervention as intended (more than 2 hours). Overall satisfaction among users of the intervention turned out to be good.

Despite a disappointing reach and compliance, some adaptations can be made to the intervention and its implementation strategy in order to increase the impact of the intervention. Together with the fact that an innovation simply needs time in order to be widely adopted [19], and the fact that, with time, a higher percentage of the population will be acquainted with the Internet and will be more skilled in using it, this gives confidence with regard to the future impact the website http://www.wiagesprek.nl can have.

## Competing interests

The authors declare that they have no competing interests.

## Authors' contributions

DB wrote the initial study protocol. DS and DB designed the intervention protocol and the website. DS wrote the manuscript, which was commented on by IS, DB, HA, WK and AB. WK had a major contribution in analyzing website user statistics. All authors have read and approved the final version of the manuscript.

## Pre-publication history

The pre-publication history for this paper can be accessed here:

http://www.biomedcentral.com/1472-6947/11/10/prepub
